# Effects of likelihood framing on side effect expectations and nocebo side effects: Results from three experimental studies with a placebo analgesic cream

**DOI:** 10.1111/bjhp.70055

**Published:** 2026-01-26

**Authors:** Tobias Kube, John M. Kelley, Arthur J. Barsky, Julia A. Glombiewski

**Affiliations:** ^1^ Department of Clinical Psychology and Experimental Psychopathology Goethe University Frankfurt Frankfurt Germany; ^2^ Department of Psychology, Pain and Psychotherapy Lab RPTU University of Kaiserslautern‐Landau Landau Germany; ^3^ Program in Placebo Studies, Harvard Medical School Beth Israel Deaconess Medical Center Boston Massachusetts USA; ^4^ Department of Psychology Endicott College Beverly Massachusetts USA; ^5^ Department of Psychiatry Brigham and Women's Hospital Boston Massachusetts USA

**Keywords:** framing, likelihood, nocebo effect, pain, placebo effect, risk perception

## Abstract

**Objectives:**

This research examined whether different framings of the likelihood of side effects influence their occurrence in response to a placebo analgesic.

**Design:**

Three independent experimental studies in non‐clinical samples were performed.

**Methods:**

Study 1 investigated the effects of likelihood framing on side effect expectations, while Studies 2 and 3 investigated the effects of likelihood framing on side effect experiences in terms of nocebo side effects.

**Results:**

Study 1 (*N* = 244) showed that participants had higher side expectations when the side effect likelihood was negatively framed (e.g. 1% occurrence) than positively framed (e.g. 99% no occurrence). Side effect expectations were also more negative when frequencies (e.g. 1 in 100) rather than percentages were used. Study 2 tested the effects of positive vs. negative framing and percentages vs. frequencies on the occurrence of itch as a side effect of a placebo analgesic cream, which was applied in an experimental heat pain paradigm in a non‐clinical sample (*N* = 179). Only 11% reported itch, with no significant differences between conditions. Study 3 (*N* = 110) increased the stated likelihood of itch, yet only 17% reported experiencing it. Crucially, reports of itch did not differ between experimental groups and a control group where itch was not mentioned as a possible side effect.

**Conclusion:**

This research failed to induce itch as a nocebo response. While examining likelihood framing in nocebo side effects is valuable, establishing an appropriate experimental model remains challenging. Future research directions and methodological considerations to move forward are discussed.

**Statement of Contribution**

**What is already known on this subject?**

- The framing of medical likelihoods influences perceived risk, even when statistics are equivalent.
- Positive framing (likelihood of a risk's non‐occurrence) is less concerning than negative framing.
- Nocebo side effects may be shaped by communicated side effect risk via expectations.

**What does this study add?**

- Likelihood framing can influence side effect expectations.
- Framing does not change the experience of itch as a nocebo side effect.

## INTRODUCTION

Research on risk communication and risk perception has demonstrated that the way in which the likelihood of a medical risk is presented significantly influences its perception (Edwards et al., 2001; Gigerenzer et al., 2007; Keller & Siegrist, 2009). Specifically, it has been shown that presenting the likelihood of an adverse medical event (e.g. side effects) occurring (‘negative framing’) leads to greater concerns than when the likelihood of it *not* occurring is presented (‘positive framing’), even though both express the same mathematical likelihood (McNeil et al., 1982; O'Connor et al., 1996; Perneger & Agoritsas, 2011). For example, people are more concerned if they are informed that there is a 1% chance of dying as a consequence of a surgery than if they are informed that there is a 99% chance of surviving it (McNeil et al., 1982). Consistent with that, recent research has shown that people are significantly more concerned when the likelihood of having a serious disease is framed negatively (‘The likelihood of having the disease is 1%’) than when it was framed positively (‘The likelihood of *not* having the disease is 99%’) (Kube et al., 2023).

In addition to positive vs. negative framing, research has also examined how the risk perception differs when a risk is presented as a numerical frequency (e.g. 1 in 100) vs. a percentage (e.g. 1%). Although research has indicated that frequencies are easier to understand (Bodemer et al., 2014; Gigerenzer et al., 2007; Gigerenzer & Galesic, 2012), there is consistent evidence that the presentation of frequencies leads to more concerns than percentages (Gurm & Litaker, 2000; Kube et al., 2023; Peters et al., 2011). Given the findings from qualitative research on how patients interpret dismal prognoses (Hagerty et al., 2005), it has been suggested that using frequencies (e.g. 1 in 100 people has a serious illness) more concretely conveys the fact that a real person is affected by the adverse event (Kube et al., 2023).

This research on the framing of medical risks can be grounded in the impactful psychological Prospect Theory (Kahneman & Tversky, 1979), which describes how people perceive and evaluate potential gains and losses relative to a reference point, rather than in absolute terms. This theory helps explain why different ways of presenting the same medical risk information can lead to significantly different patient decisions and different symptom experiences—the latter because negative framing activates loss aversion and threat sensitivity, thus increasing the likelihood of noticing bodily sensations and interpreting them negatively (e.g. as being side effects to a medication).

In recent years, the framing of medical risks has also been studied in the context of nocebo side effects, that is, the occurrence of adverse symptoms after taking a placebo (Colloca & Barsky, 2020; Colloca & Miller, 2011; Petrie & Rief, 2019). Specifically, some studies have shown that framing side effects in terms of their likelihood of occurrence (i.e. negative framing) leads to more side effects than when emphasizing the likelihood of their non‐occurrence (Barnes et al., 2019; Mao et al., 2021). Other research on framing effects for nocebo side effects has provided less clear evidence (Helfer et al., 2022; Wilhelm et al., 2018), possibly because it did not systematically vary whether the likelihoods were presented as frequencies or percentages.

The present research aimed to systematically investigate the influence of *framing* (positive vs. negative) and *likelihood presentation* (frequencies vs. percentages) on the occurrence of nocebo side effects. We addressed this in the context of an experimental heat pain paradigm, in which healthy participants were led to believe that they received an analgesic cream, although they in fact received an inert placebo cream. Besides its analgesic effects, the cream was purported to induce itch as a possible side effect. Itch was chosen because research has repeatedly demonstrated that it can be induced as a nocebo effect (Bartels et al., 2014, 2016; Napadow et al., 2015; Schut et al., 2016; Stumpf et al., 2016; van Laarhoven et al., 2011; Weng et al., 2022). Based on previous research (Barnes et al., 2019; Kube et al., 2023; Mao et al., 2021), we hypothesized that (i) negative framing leads to more nocebo itch than positive framing and (ii) that the negative framing approach leads to more itch when frequencies instead of percentages are presented, whereas we expected no such differences between the presentation of frequencies vs. percentages for positive framing. If these hypotheses were confirmed, this would be highly relevant for medical and pharmaceutical practice, as it would suggest that the likelihood of patients experiencing adverse effects after taking medication depends on how the likelihood is framed. Relatedly, if the central hypothesis regarding the beneficial effects of positive framing were confirmed, it would imply that some of the nocebo side effects in clinical practice might be prevented if clinicians were to emphasize the high likelihood of *not* having side effects, as opposed to the likelihood of having side effects.

## GENERAL METHODS

### Ethics

This research was approved by the local ethics committee of the Psychology Department of the RPTU University of Kaiserslautern‐Landau (reference number LEK_469_2023) and was conducted in accordance with the ethical standards as laid down in the 1964 Declaration of Helsinki and its later amendments. All participants gave written informed consent.

### Transparency and openness

We report how we determined our sample size, all data exclusions (if any), all manipulations and all measures in the study, and the study follows JARS (Appelbaum et al., 2018). All data, analysis code and research materials are available at https://osf.io/u6qkr/?view_only=5e1290a22ed94c8e9d459e00920e403a (or through the Appendix S1). Study 1's design and its analysis were not pre‐registered, but Studies 2 and 3 were prospectively pre‐registered as detailed in the individual study sections.

## STUDY 1

In a preliminary online study, we wanted to make sure that different framing approaches for the side effects of medication lead to different risk perceptions and expectations for the occurrence of side effects. This was important because expectations of side effects are considered an important underlying mechanism of experiencing nocebo side effects (Corsi & Colloca, 2017; Rief, Nestoriuc, et al., 2009).

### Methods

#### Participants

A non‐clinical convenience sample was recruited via email lists and social networks. Participants had to be at least 18 years old to take part in the survey, which was performed in November 2022 using the platform www.soscisurvey.de. An a priori power analysis using G*Power indicated a minimum sample of 179 participants to be able to detect a medium effect (*f* = .25) with a power of .80, α = .05, in an analysis of variance (ANOVA, between‐subjects factors) with four groups. We assumed a medium effect, as previous research found medium to large effects for the effects of positive vs. negative framing in altering people's concerns about medical risks (see above). However, since the additional aspect of frequency vs. percentage has not yet been studied in that context, we more conservatively assumed a medium effect. A total of 244 participants were included in this study. The mean age of the sample was M = 33.36 years (SD = 15.35, range: 18–78), with 79.9% female, 18.0% male, 1.2% non‐binary gender, .8% not wanting to provide information on their gender.

#### Procedure

We presented participants with vignettes about four fictional medications, each of which was said to possibly induce a specific side effect. In a between‐subject factorial design, participants were randomly assigned to one of four experimental conditions, which differed in how the likelihood of the side effects was presented: (1) negative framing—frequency, (2) negative framing—per cent, (3) positive framing—frequency and (4) positive framing—per cent. Each participant was presented with four different medications, all of which were presented using the same framing approach. We opted for manipulating the likelihood presentation in terms of a between‐subjects design instead of a within‐subjects design because we worried that presenting participants with four different medications and four different framing approaches might be confusing. Thus, this study was a mixed within‐subjects (four different medications) and between‐subjects (four different likelihood presentations) design.

The first medication vignette was an influenza vaccine, causing fever as a possible side effect, with four likelihood presentation conditions: (1) 10% chance of occurring, (2) 1 in 10 chance of occurring, (3) 90% chance of *not* occurring and (4) 9 in 10 chance of *not* occurring. The second medication was an antidepressant, leading to palpitations as a possible side effect, with four likelihood presentation conditions: (1) 1% chance of occurring, (2) 1 in 100 chance of occurring, (3) 99% chance of *not* occurring and (4) 99 in 100 chance of *not* occurring. The third medication was a painkiller, which was said to have dizziness as a possible side effect, with four likelihood presentation conditions: (1) .1% chance of occurring, (2) 1 in 1000 chance of occurring, (3) 99.9% chance of *not* occurring and (4) 999 in 1000 chance of *not* occurring. The fourth medication was a diarrhoea medication with headache as a possible side effect, with four likelihood presentation conditions: (1) .01% chance of occurring, (2) 1 in 10,000 chance of occurring, (3) 99.99% chance of *not* occurring and (4) 9999 in 10,000 chance of *not* occurring. We chose four different types of medications and side effects, and four different likelihood levels (.01% to 10% and their variants) to be able to examine how much the framing effects might generalize across different medications, side effects and likelihoods.

After each vignette, participants were asked to estimate their risk of experiencing the respective side effect (0%–100%). When doing so, participants were informed that the likelihoods that were presented to them were average numbers and that individual risk could vary based on a number of personal factors. In addition, participants were encouraged to use their own reasoning to estimate their personal risk.

#### Statistical analyses

We computed four separate 2 (negative framing vs. positive framing) by 2 (frequencies vs. percentages) analyses of variance (ANOVA), with the expected likelihood of having the side effect mentioned for each of the four vignettes as the dependent variable. Type I error levels were set at 5%. All analyses were conducted using IBM SPSS Statistics Version 29.

### Results

**FIGURE 1:**
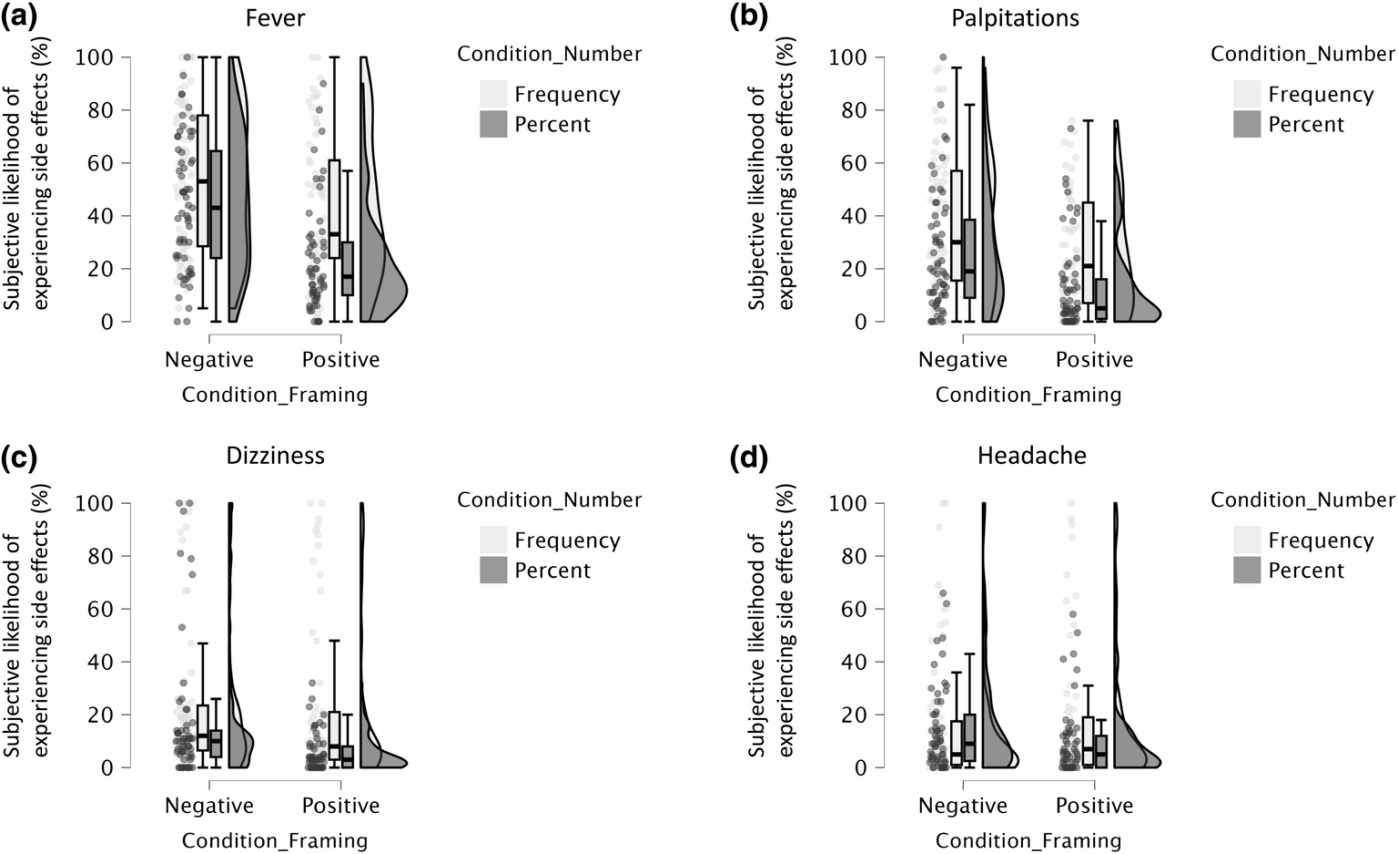
Illustration of the main results of Study 1 (raincloud plot).

For the influenza vaccine causing fever as a possible side effect, significant main effects of framing, *F*(1, 240) = 25.672, *p* < .001, *ɳ* ^*2*^_*p*_ = .097, 90% CI [.045, .158], and the likelihood presentation format, *F*(1, 240) = 21.794, *p* < .001, *ɳ* ^*2*^_*p*_ = .083, 90% CI [.035, .143], indicated that, in line with our predictions, negative framing led to a higher expected risk of experiencing side effects than positive framing and frequencies led to a higher risk than percentages (see Figure 1a).

Similarly, for the antidepressant causing palpitations as a possible side effect, significant main effects of framing, *F*(1, 240) = 19.141, *p* < .001, *ɳ* ^*2*^_*p*_ = .074, 90% CI [.029, .131] and the presentation format, *F*(1, 240) = 18.589, *p* < .001, *ɳ* ^*2*^_*p*_ = .072, 90% CI [.028, .129], indicated that negative framing led to a higher risk of experiencing side effects than positive framing and frequencies led to a higher risk than percentages (see Figure 1b).

For the pain killer causing dizziness as a side effect, significant main effects of framing, *F*(1, 216) = 8.087, *p* = .005, *ɳ* ^*2*^_*p*_ = .036, 90% CI [.006, .085], and the presentation format, *F*(1, 216) = 7.091, *p* = .008, *ɳ* ^*2*^_*p*_ = .032, 90% CI [.004, .079], indicated that negative framing led to a higher risk of experiencing side effects than positive framing and frequencies led to a higher risk than percentages, further confirming our predictions (see Figure 1c).

For headache as a side effect of the diarrhoea medication, however, neither the main effect of framing, *F*(1, 226) = 1.898, *p* = .170, *ɳ* ^*2*^_*p*_ = .008, 90% CI [0, .039], nor the main effect of the presentation format, *F*(1, 226) = .295, *p* = .587, *ɳ* ^*2*^_*p*_ = .001, 90% CI [0, .020], was significant (see Figure 1d). The framing by presentation format interaction was non‐significant for all medication vignettes.

### Discussion

This study demonstrated that for three of the four vignettes, negative framing (vs. positive framing) and frequencies (vs. percentages) were associated with a higher subjective likelihood of experiencing side effects, thereby confirming our predictions. However, contrary to our hypothesis, there was no significant interaction between framing and likelihood presentation. The results of this study suggest that the higher the likelihood of side effects, the more pronounced were the aforementioned differences between negative vs. positive framing and frequencies vs. percentages. One striking finding from this study, though, is that participants indicated personal risk scores that were significantly higher than the ‘actual’ likelihoods that were presented to them. From a methodological perspective, an important limitation is that for the fictional pain killer and the diarrhoea medication, the breadth of the scale was not sensitive enough, as the presented likelihoods were .1% and .01%, respectively, but the scale could only capture integer values (0%, 1%, 2%, etc.).

## STUDY 2

Study 1 demonstrated that side effect expectancies can be influenced by likelihood framing. Based on these results, Study 2 aimed to investigate whether different side effect *expectancies* generated by different likelihood framings would also result in different side effect *experiences*. To this end, we performed a laboratory experiment testing the four likelihood framing conditions pre‐tested in Study 1 in the context of a placebo analgesic cream, which was said to induce itch as a side effect. The hypotheses, sample size determination, analysis plan and other aspects of this study were prospectively pre‐registered at: https://aspredicted.org/16C_NYL.

### Methods

#### Participants

The sample size was determined via an a priori power analysis. We estimated the expected effect size based on previous research revealing medium effect sizes for the influence of framing on the nocebo response (Barnes et al., 2019). Accordingly, a power analysis using G*Power for a 2 by 2 ANOVA (expected effect size *f* = .25, α = .05, 1−β = .80) indicated a required minimum sample size of 179 people.

Participants were recruited via email lists and social networks at the university where the study was conducted. The study was labelled as a ‘study for the perception of heat pain’. The inclusion criteria were: age between 18 and 65 years and German language fluency. Exclusion criteria were: acute or chronic physical illness, current use of medication (except hormonal contraceptives), skin diseases, severe visual impairment, current pregnancy or breastfeeding, students of psychology from the third semester or higher (aiming to exclude students who had gained a deeper knowledge of placebo/nocebo effects). As an incentive for participation, participants received 10 EUR or course credit if they were psychology students.

Overall, 199 people participated in this laboratory experiment from May 2023 to January 2024. Of these, 20 people had to be excluded as they did not believe the cover story regarding the application of an analgesic cream (see below for the assessment of this (dis)belief). No other participant had to be excluded based on the pre‐registered criteria for excluding cases. Accordingly, all subsequent analyses are based on a sample of 179 participants randomized to the four experimental conditions (*n* = 45 for the negative framing—frequency condition, *n* = 45 for the negative framing—percentage condition, *n* = 47 for the positive framing—frequency condition and *n* = 42 for the positive framing—percentage condition). Of these, 123 participants (68.7%) identified themselves as females, 56 people (31.3%) as males, and no person indicated any other gender identity or refrained from providing information on their gender. Participants' age ranged from 18 to 43 years, with M = 22.42 years (SD = 3.08). Most participants (83.8%) had a school leaving examination (German ‘Abitur’ or ‘Fachabitur’) as the highest educational degree, 15.1% had a university degree, .6% had only a primary education and .6% were still at secondary school.

#### General procedure

The study was performed in a standard university laboratory room. The laboratory assessments were conducted by three psychology Bachelor students and two psychology Master students, based on a standardized protocol. The first author and the senior author trained the students in the study procedures.

As a part of the informed consent, participants were informed about the general procedure of the study as well as the application of the (alleged) analgesic cream. They were also informed about itching as a possible side effect of the cream, but with the presentation format of the likelihood of itching varying for the four experimental conditions (see below). Thus, each experimental condition received a slightly different version of the informed consent. This was done to increase the power of the experimental manipulation by varying the likelihood framing both at the outset in written form and subsequently as a part of the oral presentation of the (alleged) analgesic cream (see below).

After giving informed consent, participants underwent the baseline heat pain assessment, including the assessment of the individual pain threshold and pain tolerance, as detailed in the Appendix S1. Next, participants received an inert placebo cream (a standard basic cream with oil of thyme produced by a local pharmacy, as used in previous placebo studies (Kube et al., 2020; Locher et al., 2017)). All participants received the same cream. The experimenter explained the rationale for applying the cream as follows: ‘This is a commercially available pain‐relieving cream that contains a local anesthetic called lidocaine as the active ingredient. This active ingredient works very quickly and is often used for minor burns such as sunburn. Applying this cream usually makes it easier to tolerate heat pain. Do you have any questions on this?’ This explanation of the analgesic effects of the cream was the same for all participants. Subsequently, participants were informed about itching as a possible side effect of the cream. This explanation was different for the four experimental conditions (see below).

The experimenter next applied the cream on the participants' forearm before continuing with a second heat pain assessment. Participants were informed that for the cream to take full effect, it would be left to work for 2 min. This waiting period was intended to increase participants' attention to the possibility of experiencing itching after the application of the cream. The experimenter did not talk to participants during this time. Participants then completed several questions on the computer about how they perceived the application of the cream (see below). At this point, they were also asked for the first time about the degree to which they noticed itching (see below). Subsequently, participants underwent a second heat pain assessment, which was identical to the first assessment before the application of the cream. After completing the second heat pain assessment, participants completed several questionnaires (side effects, NEO‐FFI and Q‐No, see below) on the computer. As part of that assessment, participants were asked again about their degree of itching. Finally, participants were debriefed with respect to the actual purpose of the study.

#### Experimental conditions

As part of the informed consent, one experimental condition (referred to as ‘negative framing—percentage’) was presented with the likelihood of itching in the following format: ‘A possible side effect of the cream is the occurrence of itching, which occurs in about 25% of the people after applying the cream. This itching usually lasts for 30 min or less’. The first part of the first sentence as well as the second sentence were the same in the other conditions. The likelihood framings in the other three conditions were as follows: ‘…which does not occur in 75% of the people after applying the cream’ (positive framing—percentage); ‘…which occurs in 1 out of 4 people after applying the cream’ (negative framing—frequency); ‘…which does not occur in 3 out of 4 people after applying the cream’ (positive framing—frequency).

We decided on a likelihood of 25% for the following reasons. First, the likelihood of experiencing side effects had to be lower than the likelihood of not experiencing it, to be able to meaningfully compare positive and negative framing approaches. Second, the likelihood for experiencing the side effect had to be sufficiently high, so as to make sure that we could evoke nocebo side effects. If the likelihood was too low, people might not be concerned enough and, as a result, not report any side effects. The results of Study 1 also suggest that if the likelihood is too low, framing may not have an effect. Third, we wanted to choose a likelihood that can be easily translated from percentages to numerical frequencies. Based on these considerations, we opted for a likelihood of 25%, but we acknowledge that with the same reasoning, we could also have chosen 20% or 33%.

In addition to the informed consent, likelihood framing was manipulated as part of the oral explanation provided for the application of the (alleged) analgesic cream after the first heat pain assessment. Specifically, after participants were informed about the analgesic effects of the cream, they were informed about its possible side effects. The experimenter first explained: ‘However, a possible side effect of this analgesic cream is itching’. This sentence was stated in all experimental conditions. Next, the experimenter indicated the likelihood of experiencing (or not experiencing) side effects based on the experimental condition to which participants were assigned. The exact wording for the four experimental groups is presented in Table 1.

**TABLE 1:** Presentation of the four different likelihood presentation approaches.

	Negative framing	Positive framing
Frequencies	‘Itching occurs in 1 out of 4 people, who apply this cream’	‘Itching does not occur in 3 out of 4 people, who apply this cream’
Percentages	‘Itching occurs in 25% of the people, who apply this cream’	‘Itching does not occur in 75% of the people, who apply this cream’

Of note, we did not include a control condition receiving no information regarding itch because previous research has repeatedly shown that verbal suggestions, such as mentioning the likelihood of experiencing itch as a response to medical procedures (which in fact were sham stimulations which could not directly produce itch), reliably induce nocebo itch (Bartels et al., 2014; Schut et al., 2016; Stumpf et al., 2016; van Laarhoven et al., 2011). Thus, we were confident that the verbal suggestion regarding the occurrence of itch would induce the experience of itch, so we decided to maximize power for the comparison of the different framing variants.

#### Randomization and blinding

Prior to each experimental session, participants were assigned by the experimenter to one of the four conditions. To do so, the investigator drew a number (1 = negative framing—per cent; 2 = positive framing—per cent; 3 = negative framing—frequency; 4 = positive framing—frequency) out of a concealed envelope. In addition, it was randomized on which location of their forearm participants started the heat pain assessment (see Appendix S1). The experiment was single‐blinded (i.e. the experimenter was aware of the conditions to which the participants were randomly assigned, but participants were kept blind to the four experimental conditions).

#### Measures

##### Side effects

Our pre‐defined primary outcome was the extent to which participants reported itching on their forearms after applying the cream. This was assessed by using the itching item from the Generic Assessment of Side Effects (GASE) scale (Rief, Glombiewski, & Barsky, 2009). Using a 4‐point scale, participants indicated the extent to which they experienced itching on their forearm (from 1 = ‘not at all’ to 4 = ‘very much’). This was assessed twice: 2 min after applying the cream and after completing the post‐treatment heat pain assessment.

If participants indicated that they experienced at least some itching on the aforementioned scale, we additionally assessed the degree to which participants were bothered by itching (from 1 = ‘not at all’ to 4 = ‘very much’) and how often they felt the urge to scratch themselves (from 1 = ‘not at all’ to 4 = ‘all the time’).

##### Expected side effects

Similar to previous nocebo studies, we assessed the degree to which participants expected to experience side effects. To this end, participants were asked the following question: ‘How likely do you think it is that you will experience itching as a side effect of the cream?’ and indicated their response on a scale from 0% to 100%. In addition, participants indicated the expected intensity of itching (from 1 = ‘not at all’ to 4 = ‘extremely intense’) and the degree to which they worried about the occurrence of side effects (from 1 = ‘not at all’ to 4 = ‘very much’).

##### Treatment expectancies

As with previous studies (Friehs et al., 2022, 2024), we used the 5‐item treatment expectancies scale to assess the degree to which participants expected the cream to help them cope with the painful stimulations (Kube et al., 2020). Each item was assessed using a 5‐point Likert scale, with higher values reflecting higher treatment expectancies.

##### Credibility of the cover story

To assess the credibility of the cover story regarding the alleged analgesic cream, we used the same questions as previous research (Kube et al., 2020; Locher et al., 2017). Specifically, participants were asked to rate whether they believed they had received an analgesic cream (from 1 = ‘I was sure that I received a pain‐killing cream’, 2 = ‘I doubted whether I received a pain‐killing cream’ and 3 = ‘I did not believe that I received a pain‐killing cream’). As with previous research, we excluded participants who did not believe that they had received an analgesic cream (i.e. scoring 3 on the aforementioned scale). We also repeated the analyses after excluding those participants who reported some doubts about the receipt of an analgesic cream (*n* = 55), but this did not lead to a different pattern of results as compared to the main results presented below.

##### Other measures

To assess possible moderating variables, we assessed neuroticism and openness to new experiences, using the appropriate items from the NEO Five Factor Inventory (NEO‐FFI) and coping with side effects to previous medications, using the Q‐No (Mitsikostas & Deligianni, 2015). These variables were chosen because personality traits such as neuroticism and openness have been shown to influence the placebo response (Davis et al., 1995; Hyland et al., 2007). Additionally, sociodemographic variables, including age, gender and education level, were obtained.

#### Statistical analyses

First, we conducted data screening according to the suggestions made by Tabachnick et al. (2007) and tested the assumptions of analysis of variance (ANOVA). There were no missing values due to the study design (participants could only continue if they entered all values). For the pre‐registered main analysis, we computed two separate 2 (negative framing vs. positive framing) by 2 (frequencies vs. percentages) ANOVAs, with the amount of itching in the last few minutes vs. after the last pain assessment, as assessed with the GASE, as the dependent variable. Although not pre‐registered, we followed the journal policy to report Bayesian analyses for non‐significant results regarding the significance tests of the core hypotheses. In addition, we computed Pearson correlations for the associations of itching with several psychological constructs. Furthermore, we explored group differences in pain perception via analyses of covariances (ANCOVA) using the post‐treatment variable of pain perception (pain threshold, pain tolerance and corresponding intensity ratings) as the dependent variable and the respective pre‐treatment variable as a covariate. As noted in another related preregistration (https://aspredicted.org/hsjc‐2py2.pdf), we will examine and report elsewhere if the experience of side effects predicts changes in perceived pain intensity following the placebo cream. Type I error rates were set at 5%. The main analyses were conducted using IBM SPSS Statistics (version 29) and the Bayesian analyses were conducted using JASP (version 0.19).

### Results

#### Group differences in expected side effects

**FIGURE 2:**
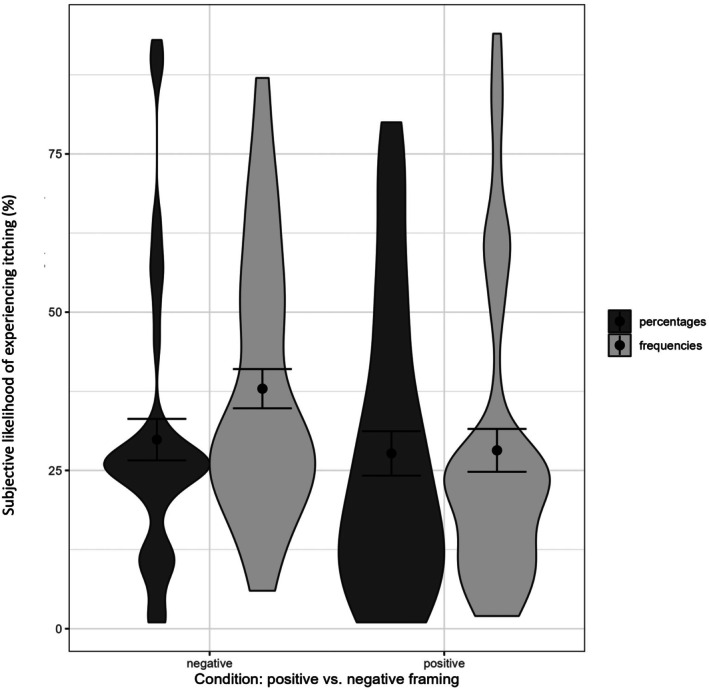
Violin plot of the group differences in expected side effects. Error bars reflect the 95% confidence interval.

An ANOVA indicated neither the main effect of framing, *F*(1, 175) = 3.213, *p* = .075, *ɳ* ^*2*^_*p*_ = .018, 90% CI [0, .063], nor the main effect of presentation format, *F*(1, 175) = 1.644, *p* = .201, *ɳ* ^*2*^_*p*_ = .009, 90% CI [0, .046], nor the framing by presentation format interaction, *F*(1, 175) = 1.295, *p* = .257, *ɳ* ^*2*^_*p*_ = .007, 90% CI [0, .042] were significant. For the main effect of framing, though, there was a small non‐significant trend suggesting that on a descriptive level negative framing was associated with a somewhat higher estimated likelihood than positive framing (see Figure 2). Specifically, the ‘negative framing—frequency’ condition (M = 37.91, SD = 20.81) reported somewhat higher likelihoods than the ‘negative framing—percent’ condition (M = 29.87, SD = 22.00), the ‘positive framing—frequency’ condition (M = 28.17, SD = 23.24) and the ‘positive framing—percent’ condition (M = 27.69, SD = 22.74). This descriptive trend is at least partially consistent with the results from Study 1. The experimental conditions did not significantly differ in the expected intensity of itching and their degree of worry about it either (all *p*‐values > .171). Thus, contrary to the results of Study 1, the manipulation of the likelihood presentation in this study failed to induce significantly different cognitive‐affective responses. The non‐preregistered Bayesian analyses for side effect expectancies are reported in Table S1. Bayes factors for inclusion indicated evidence against all three effects: framing (BF_incl = .53; equivalently BF_excl = 1.89, anecdotal evidence against), presentation format (BF_incl = .26; BF_excl = 3.85, moderate evidence against) and the framing by format interaction (BF_incl = .17; BF_excl = 5.88, moderate evidence against).

#### Group differences in reported side effects

##### Descriptive report of side effect

Overall, only a minority of the sample reported itching as a side effect of the cream. Specifically, 10.6% of the participants indicated that they experienced mild itching in the last few minutes after applying the cream. After the post‐treatment pain assessment, 11.7% indicated that they experienced mild itching in the last 30 min, and only one person reported a moderate amount of itching at this point. The degree of reported itching in the last few minutes correlated significantly with the degree of itching in the last half hour, *r* = .649, *p* < .001. Only 3.9% (5.6% in the last 30 min, respectively) reported that they were mildly bothered by itching, and 2.2% (1.1% in the last 30 min, respectively) reported the urge to scratch themselves from time to time, while one person reported having had this urge most of the time. Thus, only a few people reported itch as a nocebo side effect of the cream, resulting in limited variance of the primary outcome variable.

##### Differences between the experimental conditions in reported side effects

**FIGURE 3:**
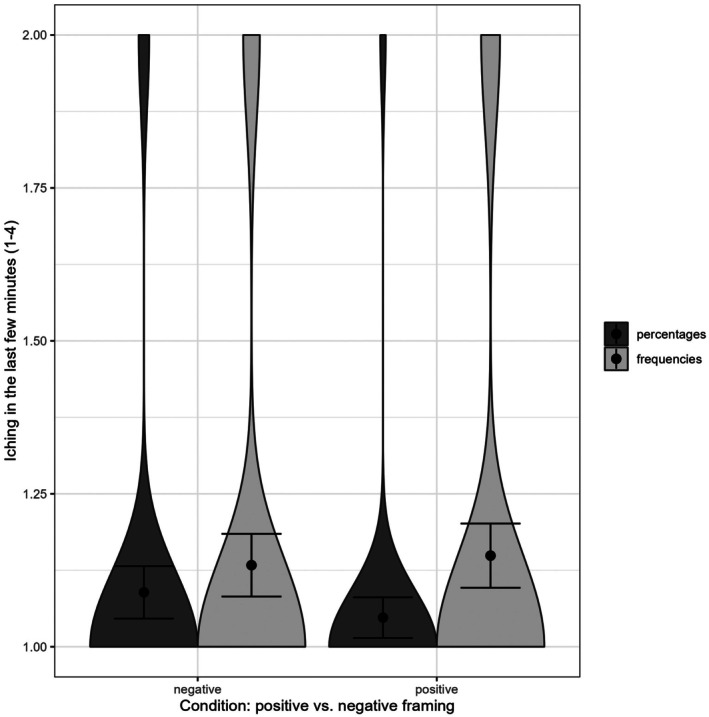
Violin plot of the group differences in reported side effects. Error bars reflect the 95% confidence interval.

The ANOVA considering the degree of itching in the last few minutes as the dependent variable indicated that neither the main effect of framing, *F*(1, 175) = .077, *p* = .782, *ɳ* ^*2*^_*p*_ < .001, 90% CI [0, .016], nor the main effect of the presentation format, *F*(1, 175) = 2.486, *p* = .117, *ɳ* ^*2*^_*p*_ = .014, 90% CI [0, .055], nor the framing by presentation format interaction, *F*(1, 175) = .378, *p* = .539, *ɳ* ^*2*^_*p*_ = .002, 90% CI [0, .028] were significant (see Figure 3 for an illustration). Bayes factors for inclusion indicated evidence against all three effects for this outcome: framing (BF_incl = .121; equivalently BF_excl = 8.26, moderate‐to‐strong evidence against), format (BF_incl = .354; BF_excl = 2.82, anecdotal‐to‐moderate evidence against) and the framing by format interaction (BF_incl = .062; BF_excl = 16.13, strong evidence against); see Table S2. Overall, across the model space, the data were several times more likely under models excluding each effect than under models including them. The results did not significantly differ when considering itching in the last 30 min instead of the last few minutes.

#### Associations of nocebo itching with other variables

Across experimental conditions, those participants who expected to have a high likelihood of experiencing itching actually reported having more itching in the last 30 min (*r* = .278, *p* < .001) and in the last few minutes, respectively (*r* = .205, *p* = .006). Consistent with that, participants who scored higher on neuroticism reported significantly more itching in the last few minutes (*r* = .177, *p* = .018), but this effect did not hold for itching in the last 30 min (*r* = .117, *p* = .120). Reports of itching were unrelated to openness to new experiences, attitudes towards medication and treatment expectancies (all *p*‐values > .139). All correlations are presented in Table 2. Note that these correlational analyses were not pre‐registered.

**TABLE 2:** Correlations among reported itching and psychological variables.

	Itching—last 30 min	Itching—last few min	Expected likelihood of having itching	Treatment expectancies	Neuroticism	Openness to new experiences	Attitudes towards medication
Itching—last 30 min	–	.649***	.278***	−.024	.117	−.087	.111
Itching—last few min	–	–	.205**	−.127	.177*	−.089	.097
Expected likelihood of having itching	–	–	–	−.063	.153*	−.062	.060
Treatment expectancies	–	–	–	–	−.080	−.018	.149*
Neuroticism	–	–	–	–	–	.031	.062
Openness to new experiences	–	–	–	–	–	–	.160*

*Note*: *p < .05, **p < .01, ***p < .001.

### Discussion

This study failed to find the hypothesized differences in nocebo itching based on how the likelihood of occurrence was framed. Since the number of participants who reported itching after applying the cream was very low, a plausible explanation of this failure is that limited variance in nocebo itching made it very difficult to find significant differences between the different framing conditions. Relatedly, it might be that the communicated likelihood of itching (25% and its different framing variants, respectively) was too low to produce nocebo side effects. Alternatively—and more fundamentally—it is possible that itching as a side effect of a purported analgesic cream is simply not a good model to investigate the effects of framing on nocebo side effects. This interpretation is somewhat complicated, though, by the fact that there was no control condition receiving no information regarding itching as a potential side effect of the cream.

## STUDY 3

To address the aforementioned limitations of Study 2, we performed another experiment, in which we (i) aimed to enhance the occurrence of itching by using a higher communicated likelihood of occurrence and (ii) added a control condition receiving the cream without any information on itching.

### Methods

#### Experimental design

The basic procedure was the same as in Study 2. However, the verbal suggestions for nocebo itching were different, resulting in different experimental conditions. In particular, the likelihood of itching occurring as a side effect of the analgesic cream was increased from 25% to 60%. In one condition, this was mentioned as a percentage, referred to as the *percentage* condition. The entire instruction read: ‘Itching is a common side effect of this cream. This occurs in 60% of people who have used this cream. Itching can start immediately after applying the cream, but usually does not last longer than half an hour. Other reported side effects included dizziness, nausea, skin irritations and mild numbness, but these occurred very rarely’. In the second condition, the instruction was the same, but the likelihood of itching was presented as a frequency (‘This occurs in 6 out of 10 people who have used this cream’), referred to as the *frequency* condition. In addition, there was a control condition, in which itching was not mentioned as a possible side effect. Instead, the control condition received the following information: ‘There have been a few reports of dizziness and mild numbness as side effects of the cream, but these occurred very rarely’.

The rationale behind these changes in the experimental design was as follows: Our primary intention was to include a control condition receiving no information on itching, such that we could compare the possible occurrence of itching in the experimental conditions with the base rate of itching after applying the placebo cream. Accordingly, in the control group, itching was not mentioned as a potential side effect, but instead, we mentioned two other possible side effects (dizziness and numbness) because we reasoned that it would appear implausible for participants if an analgesic cream was claimed to have no side effects at all.

For the experimental conditions, we decided to focus on the comparison of frequency vs. percentage (instead of positive vs. negative framing or the entire 2 by 2 factorial design from Study 1), because there was a small descriptive trend in Study 2 showing slightly more itching in the frequency condition than in the percentage condition, whereas the framing factor (positive vs. negative) showed essentially zero effect. Furthermore, as we aimed to increase the presented likelihood of itch above 50% to enhance the chance to induce nocebo side effects, the meaning of the negative vs. positive framing would have changed with the aversive event being more likely to occur than not to occur. Thus, we decided to focus on two conditions in which the likelihood of the occurrence of itch as a side effect is mentioned, which is how information on side effects of medication is typically presented to patients. We chose to set this likelihood at 60% rather than the previously used 25% to enhance the chance of inducing nocebo side effects. On the other hand, it must not be too high, so as to leave some room for uncertainty, such that participants' concerns and worries about the possible occurrence of side effects can come into play.

#### Participants

Participants were recruited as in Study 2, using the same inclusion and exclusion criteria. As an incentive for participation, participants received 15 EUR as financial compensation or course credit if they were psychology students.

For this experiment, we performed a power analysis for a three‐group experiment to be analysed in a univariate ANOVA. This power analysis was based on the goal to find significant differences between the experimental conditions and the control group in the experience of itching. In other words, the sample size should offer sufficient power to detect a nocebo side effect. Since previous research usually found medium to large effects in terms of the mere demonstration of nocebo effects in experimental groups as compared to the control groups (Colloca & Barsky, 2020; Petrie & Rief, 2019; Rief et al., 2011), we expected to find an effect of *f* = .32, and a power analysis using G*Power (α = .05, 1−β = .80) indicated a minimum sample size of 98 participants. Overall, 120 people participated in this laboratory experiment from May 2023 to September 2023. Of these, 10 people had to be excluded as they did not believe the cover story regarding the application of an analgesic cream (which was assessed as in Study 2). Accordingly, all subsequent analyses are based on a sample of 110 participants, with *n* = 37 for the frequency condition, *n* = 37 for the percentage condition and *n* = 36 in the control condition.

Of these, 80 people (72.7%) identified themselves as females, 30 people (27.3%) as males, and no person indicated any other gender identity or refrained from providing information on their gender. Participants' age ranged from 18 to 65, with 58.2% being between 20 and 24 years old and 28.2% between 25 and 29 years.^1^ Most participants (69.1%) had a school leaving examination (German ‘Abitur’ or ‘Fachabitur’) as the highest educational degree, 27.3% had a university degree, 2.7% had completed their professional training and .9% had primary education. Most participants (81.8%) were university students, 10.9% were employed, 2.7% were in professional training, 1.8% were at a secondary school and 1.8% were unemployed.

#### Measures

The measures were the same as in Study 2, but here we added the items from the GASE to assess dizziness, nausea, skin irritations and numbness. For each of the symptoms, participants indicated the extent to which they experienced the symptom in the last few minutes after applying the cream and in the last 30 min (after completing the last pain assessment). As compared to Study 2, we decided to increase the breadth of the scale of the GASE items from 1–4 to 1–10 because the sensation of itch might have been too weak to be captured by a 4‐point scale but might be captured by a 10‐point scale (1 = ‘not at all’, 10 = ‘very much’).

#### Statistical analyses

The approach for the statistical analyses was basically the same as in Study 2, but since there were only three experimental groups, the main analysis was a univariate ANOVA with the experimental conditions as the independent variable and the extent of itch as the dependent variable.

### Results

#### Group differences in expected side effects

An ANOVA indicated that the three experimental conditions differed significantly in their estimated likelihood of experiencing itching as a side effect of the cream, *F*(2, 107) = 10.419, *p* < .001, *ɳ* ^*2*^_*p*_ = .163, 90% CI [.061, .258]. Pairwise comparisons indicated that both the *frequency* condition (M = 41.73, SD = 26.73), *t*(71) = 2.950, *p* = .004, *d* = .691, 95% CI [.216, 1.161] and the *percentage* condition (M = 51.03, SD = 25.49), *t*(71) = 4.727, *p* < .001, *d* = 1.107, 95% CI [.610, 1.597] produced significantly higher values for the expected likelihood of itch than the control condition (M = 25.03, SD = 21.25). However, the frequency and the percentage condition did not differ significantly, *t*(72) = 1.531, *p* = .130, *d* = .356, 95% CI [−.105, .814]. Regarding the post‐hoc comparison of the two experimental conditions, a Bayes Factor of BF_10, U_ = .656 indicates weak/anecdotal evidence in favour of no difference between that particular pair of conditions.

The three groups significantly differed in the expected intensity of itch, *F*(2, 107) = 7.908, *p* < .001, *ɳ* ^*2*^_*p*_ = .129, 90% CI [.038, .220]. Participants from the *percentage* condition expected itch to be significantly more intense than participants from the frequency condition, *t*(72) = 2.316, *p* = .023, *d* = .538, 95% CI [.073, 1.001] and the control condition, *t*(65.813) = 4.004, *p* < .001, *d* = .933, 95% CI [.447, 1.414]. The *frequency* condition and the control group did not significantly differ in the expected intensity of itch, *t*(68.313) = 1.561, *p* = .123, *d* = .364, 95% CI [−.100, .826]. The three experimental conditions also differed significantly in the extent to which they worried about the occurrence of itch, *F*(2, 107) = 3.978, *p* = .022, *ɳ* ^*2*^_*p*_ = .069, 90% CI [.006, .147], with the *percentage* condition being significantly more worried than the control condition, *t*(67.982) = 2.764, *p* = .007, *d* = .645, 95% CI [.172, 1.114], while the other comparisons were not significant (*p* > .105).

The three experimental conditions did not differ in terms of their estimated likelihood of experiencing other side effects (all *p*‐values > .101).

#### Group differences in reported side effects

##### Descriptive report of side effects

In this study, 17.3% of the participants indicated that they experienced mild itching in the last few minutes after applying the cream (14.5% after the last pain assessment). Only 10.0% indicated that they were mildly or moderately bothered by itching (5.5% after the last pain assessment) and 5.5% (8.2% after the last pain assessment) reported the urge to scratch themselves. Thus, although the occurrence of itch was slightly higher than in Study 1 (17.3% vs. 10.6%), the majority of participants did not report itching as a nocebo side effect of the cream, again resulting in limited variance in the primary outcome variable.

As for the other side effects, 9.1% reported mild dizziness in the last few minutes (12.7% after the last pain assessment), 3.6% reported nausea (both in the last few minutes and after the last pain assessment), 4.5% reported minor skin irritations (8.2% after the last pain assessment) and 24.5% reported mild to moderate numbness at the location where the cream was applied (25.5% after the last pain assessment).

##### Differences between the experimental conditions in reported side effects

**FIGURE 4:**
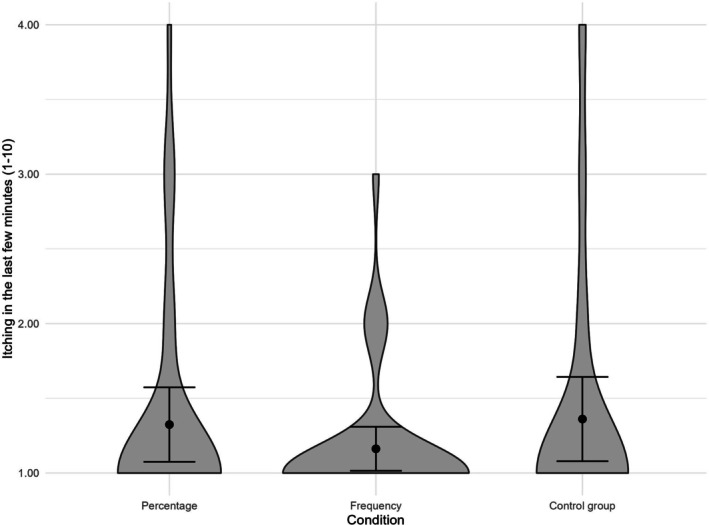
Violin plot of the group differences in reported side effects. Error bars reflect the 95% confidence interval.

The ANOVA indicated that the three experimental conditions did not significantly differ in the occurrence of itching in the last few minutes, *F*(2, 107) = .856, *p* = .482, *ɳ* ^*2*^_*p*_ = .016, 90% CI [0, .061], see Figure 4. A Bayesian ANOVA provided moderate evidence against an effect of the experimental condition (BF_incl = .171; equivalently BF_excl = 5.85), indicating that the data were substantially more likely under models excluding the condition factor than under models including it, see Table S3. When considering itching in the last 30 min as the dependent variable, the experimental conditions did not differ either, *F*(2, 107) = .898, *p* = .410, *ɳ* ^*2*^_*p*_ = .017, 90% CI [0, .063]. Thus, since the two experimental groups did not report more itch than the control group, the study failed to induce itch as a nocebo side effect. The experimental conditions did not differ in reported dizziness, skin irritations or numbness either (all *p*‐values > .146). However, the groups did differ significantly in the degree of reported nausea, *F*(2, 107) = 4.304, *p* = .016, *ɳ* ^*2*^_*p*_ = .074, 90% CI [.008, .154], with the *percentage* condition reporting significantly more nausea than the *frequency* condition (*t*(36) = 2.089, *p* = .040, *d* = .486, 95% CI [.022, .947]) and the control condition (*t*(36) = 2.089, *p* = .044, *d* = .482, 95% CI [.015, .946]). Yet, it should be noted that this effect was driven by only four participants reporting some nausea who happened to be in the *percentage* condition, whereas the remaining 106 participants did not report any nausea.

### Discussion

This study suggests that even with a higher communicated likelihood of occurrence (60% vs. 25%), only very few people reported itching after the application of the alleged analgesic cream. Since the two experimental groups did not report more itching than the control group, the study failed to induce itching as a nocebo side effect. Possible interpretations of this failure and implications for future research are discussed below. Of note, although participants from the percentage condition reported significantly more nausea than participants from the other conditions, we believe that this is most likely a Type I error. If it was a real nocebo side effect, this should have been reflected by a higher expected likelihood of occurrence of nausea in that condition, accompanied by a higher level of worries, neither of which was the case. Therefore, given the very few people who reported nausea and the lacking theoretical connection between the instructions and the experience of nausea, we believe this was merely a chance finding.

## GENERAL DISCUSSION

Previous research demonstrated that people respond very differently to medical outcome likelihoods depending on how they are framed (Barnes et al., 2019; Kube et al., 2023; McNeil et al., 1982), as predicted by the influential Prospect Theory (Kahneman & Tversky, 1979). The present research built on this established body of literature to investigate whether framing effects can influence the rate of nocebo side effects to a supposedly analgesic cream. This issue is not only of academic interest, but also of potential practical importance given the well‐known effect of negative side effect expectations on the experience of side effects (Corsi & Colloca, 2017; Petrie & Rief, 2019; Rief, Nestoriuc, et al., 2009).

The results of Study 1 demonstrated that when presenting participants with fictitious medication vignettes, different framing approaches can indeed lead to different estimations of the personal likelihood of having side effects. In particular, in line with our predictions and previous research (McNeil et al., 1982; O'Connor et al., 1996; Perneger & Agoritsas, 2011), the results showed that negative framing (i.e. presenting the likelihood of having a side effect) evoked higher subjective likelihood estimations than positive framing (i.e. presenting the likelihood of *not* having a side effect). In addition, presenting the likelihood of a side effect as a frequency led to a higher subjective likelihood of experiencing side effects than the presentation of percentages. In Study 2, however, when the likelihood presentation was embedded into an actual pain assessment procedure, the same framing variations (positive vs. negative framing and frequencies vs. percentages) did not lead to significantly different responses. There was only a small descriptive trend, suggesting that negative framing is associated with a higher perceived likelihood of having a side effect, consistent with Study 1. Importantly, the results of Study 2 show that the four different framing approaches did not influence the occurrence of nocebo itching. Study 3 suggested that the negative results of Study 2 might have arisen because it is not possible to induce a significant amount of itching as a nocebo side effect to an analgesic cream through these sorts of verbal suggestions. Specifically, the results of both Study 2 and Study 3 indicated that the number of people who reported itching was low, and Study 3 showed that the occurrence of itching in the experimental groups was not higher than in the control group. Thus, we have to conclude that the Studies 2 and 3 could not achieve their aim to systematically investigate the influence of likelihood framing on the occurrence of nocebo side effects because it was not possible to induce nocebo side effects.

Several explanations may account for this failure. First, it is conceivable that our experimental model, which focused on the occurrence of itching as a side effect of a putative analgesic cream, was not suitable to evoke nocebo side effects. We chose this model because previous research had consistently shown that itching can be reliably induced as a nocebo side effect following sham medical procedures (Bartels et al., 2014, 2016; Napadow et al., 2015; Schut et al., 2016; Stumpf et al., 2016; van Laarhoven et al., 2011; Weng et al., 2022). However, according to a recent review of experimental methods to study nocebo side effects on itch, most previous studies using verbal suggestions applied histamine, electrical or mechanical stimulations to evoke itch (Blythe et al., 2019), whereas the present study was the first to introduce itch as a potential side effect to an analgesic cream. Related to this point, itching is often treated with creams and creams tend to be soothing, so it might be that the physicality of this cream and its associated non‐verbal meaning overwhelmed or contradicted the verbal suggestion that it could cause itching. In addition, topical analgesics (such as Lidocaine, which was the purported ingredient of the cream used in Studies 2 and 3) not only treat pain, but they can also relieve itching. Although this was not mentioned in the instructions provided to the participants, some participants might have been aware of this, so that for them the suggestion of itching as a side effect might have been at odds with their prior experiences or knowledge. Yet, no participant mentioned this explicitly in the follow‐up interview when asked about the plausibility of the cover story.

Taking these issues regarding the choice of our experimental model into account, future research may either have to use other itch‐evoking methods or focus on other potential side effects of a placebo analgesic cream. The results from Study 3 suggest that numbness might be a possible target in that respect, since this was the most frequently reported side effect. However, numbness could also be a direct (and in fact desired) effect of the cream. In addition, numbness was reported by about 25% of the participants, which might still be too low to ensure enough variance for detecting effects of different framing approaches.

Furthermore, it is possible that the verbal suggestions did not work well enough to induce nocebo side effects. In fact, the prerequisite for showing the influence of different likelihood framings on the occurrence of nocebo side effects is that they evoke different cognitive‐affective responses. However, the results of both Study 2 and Study 3 show that there was not much difference in how the experimental groups perceived the different framing variants. This was surprising because Study 1 did show significant differences. A possible explanation for why these differences did not hold in the laboratory studies is that in Study 1, the focus from the participants' perspective was solely on the introduction of possible side effects of various medications. In Studies 2 and 3, by contrast, this was just one aspect, which was embedded into a much broader procedure. In fact, the study was labelled as a study on pain perception (to reduce demand effects) and many participants concentrated on how much pain they could tolerate. Accordingly, it might be that the introduction of the possible side effects of the placebo analgesic cream was not salient enough given the entire procedure. Hence, future research may have to be more explicitly centred on the introduction of the likelihood of side effects.

With regard to the precise wording of the verbal suggestions in Study 2, we note that from an experimental viewpoint, we aimed to keep the wording of the positive framing as similar as possible to the negative framing condition. However, from an efficacy perspective as well as an ecological validity perspective, it might have been preferable to convey a more positive message, such as, ‘A possible side effect of the cream is the occurrence of itching, but the good news is that 3 out of 4 people do not experience this side effect’. Such a wording may be considered if the goal was to optimize side effect expectancies.

Another related methodological aspect to consider might be that in Study 1, participants were informed that the side effect information presented are average reports and their individual likelihood might differ from that. Related to this point, participants from Study 1 were encouraged to use their own thinking when estimating their subjective likelihood of having a side effect. This encouragement to reflect on the subjective likelihood of having side effects was lacking in Studies 2 and 3 because we worried that it might raise doubts about the cover story. Yet, it might be that with the more subtle procedures of Studies 2 and 3, the salience of side effects was just not high enough for different framing approaches to produce significant effects.

Moreover, two of the major psychological mechanisms underlying nocebo effects are symptom misattribution and symptom amplification (Colloca & Barsky, 2020; Rief et al., 2011). That is, symptoms that might have otherwise been experienced anyway (e.g. headaches, muscle aches or indeed, itchiness) are misattributed to a treatment and/or are amplified by increased attention and concern being directed at those symptoms arising from the knowledge that the symptoms might be side effects of the treatment. In the experimental paradigm used in Studies 2 and 3, it is relatively unlikely that participants would be coming into the lab with noticeable itchiness, and therefore in the absence of a pre‐existing feeling of itchiness, there is nothing to misattribute or amplify. Relatedly, a possible direction of future research on framing effects could be to focus on side effects of active medications (or other medical treatments), as was successfully done in some previous studies (O'Connor et al., 1996). If future research were to take some of the possible directions outlined above, it can not only inform psychological theory building (e.g. prospect theory) but also provide suggestions for clinical practice, with the aim of reducing nocebo effects while ensuring informed consent at the same time.

### Limitations and strengths

As discussed above, the major limitation of the present research is that we were not able to establish an experimental model to induce nocebo side effects. In addition, we note that the clinical relevance of the findings is limited given the experimental setting and the non‐clinical sample. Further limitations regarding the samples arise from the low degree of diversity in them, as both studies examined relatively young, well‐educated and largely female samples. Furthermore, in terms of statistical power, we note that Studies 2 and 3 were probably underpowered since we based our a priori power analyses on the assumption that nocebo side effects could be induced based on prior research (see above for our detailed reasoning). However, in view of the current results, this assumption was too optimistic.

Notwithstanding these limitations, the merit of this research can be seen in the conduction of three consecutive experimental studies, in which the effects of positive vs. negative framing and the presentation of percentages vs. frequencies were examined, both in an online vignette study (Study 1) and laboratory experiments (Studies 2 and 3). Further strengths can be seen in the open availability of the data and the analysis code as well as the preregistration of the hypotheses and the analysis plan of Study 2.

## CONCLUSIONS

The present research systematically varied the use of different framing approaches when informing participants about the likelihood of side effects occurring after applying a placebo analgesic cream. The results show that the choice of an experimental heat pain model and the application of a placebo analgesic cream to evoke itch as a side effect are not well‐suited to investigate these framing effects. To better examine the effects of likelihood framing on the occurrence of nocebo side effects, other methodological approaches are required.

## AUTHOR CONTRIBUTIONS

**Tobias Kube:** Conceptualization; investigation; writing – original draft; writing – review and editing; visualization; validation; methodology; software; project administration; formal analysis; resources; supervision; data curation. **John M. Kelley:** Conceptualization; methodology; supervision; writing – review and editing. **Arthur J. Barsky:** Conceptualization; methodology; writing – review and editing; supervision. **Julia A. Glombiewski:** Conceptualization; investigation; writing – review and editing; supervision; methodology.

## FUNDING INFORMATION

The present research received no funding.

## CONFLICT OF INTEREST STATEMENT

The authors have no conflict of interest to declare.

## Supporting information


Appendix S1

## Data Availability

The data that support the findings of this study are openly available in Open Science Framework at https://osf.io/u6qkr/?view_only=5e1290a22ed94c8e9d459e00920e403a.

## Footnotes

^1^
Since participants in this study were asked their age in categories of 5 years each (e.g., 20–24, 25–29, etc.), the mean and SD cannot be computed.
