# Supplementary material for: Effects of likelihood framing on side effect expectations and nocebo side effects: Results from three experimental studies with a placebo analgesic cream
Source: Br J Health Psychol. 2026 Jan 26;31(1):e70055. doi: 10.1111/bjhp.70055 (PMC12835575; doi:10.1111/bjhp.70055)
Supplement: Supplementary file 1 — Appendix S1 [file BJHP-31-0-s001.docx]

**Supplementary Material**

**Heat pain assessments**

For the pain assessments, we used the suprathreshold method of the Thermo Sensory Analyser (TSA-II), a commonly used device to examine pain in an experimental setting. To prevent the effects of sensitization or habituation (Emerson et al., 2014), the thermode of the TSA-II was fixed on two different locations on the non-dominant forearm (A and B). For half of the participants (according to randomization), the thermode was first applied to position A in the baseline pain assessment, followed by position B for the post-treatment assessment. For the other half, it was vice versa. Prior to beginning with the actual measurements, participants were made familiar with the thermode, without applying painful stimulations.

The baseline heat pain assessment started with assessing participants’ heat pain threshold (i.e., the point when sensation changes from being warm to being painful). We used the method of limits, starting at 32°C with a rise of 0.5°C every second. Participants were instructed to stop the temperature increase by pressing any button of the computer mouse in front of them. To prevent physical injuries, the measurement stopped automatically when the maximum temperature of 51°C was reached, unless participants stopped it beforehand. Participants were asked about how intense the pain was when they stopped the temperature increase (on a scale from 0-100, see below). Subsequently, the temperature immediately declined to the initial adaptation temperature of 32°C (slope 10°C/second). This procedure was repeated three times, to be able to control for normal fluctuations in participants’ heat pain threshold, in line with previous research (Kube et al., 2020; Locher et al., 2017). After assessing participants’ pain threshold, participants’ heat pain tolerance was assessed. Here, participants were asked to stop the temperature increase when they could not stand the thermal stimulations any longer. Again, this would have stopped automatically upon reaching 51°C if participants had not stopped it themselves. As with pain threshold, participants were asked how intense the painful sensation was when they stopped the temperature increase. Similar to pain threshold, the procedure to examine participants’ pain tolerance was performed three times.

**Additional Results**

**Study 2**

***Bayesian ANOVA for side effect expectancies***

Table S1

| *Analysis of Effects – Side Effect Expectancies* | | | | | | | | | | | |
| --- | --- | --- | --- | --- | --- | --- | --- | --- | --- | --- | --- |
| Effects | | P(incl) | | P(excl) | | P(incl\|data) | | P(excl\|data) | | BF_incl_ | |
| Condition_framing |  | 0.600 |  | 0.400 |  | 0.442 |  | 0.558 |  | 0.527 |  |
| Condition_format |  | 0.600 |  | 0.400 |  | 0.282 |  | 0.718 |  | 0.261 |  |
| Condition_framing ✻  Condition_format |  | 0.200 |  | 0.800 |  | 0.041 |  | 0.959 |  | 0.173 |  |
|  | | | | | | | | | | | |

***Bayesian ANOVA for side effect experiences***

Table S2

| *Analysis of Effects – Itching in the Last Few Minutes* | | | | | | | | | | | |
| --- | --- | --- | --- | --- | --- | --- | --- | --- | --- | --- | --- |
| Effects | | P(incl) | | P(excl) | | P(incl\|data) | | P(excl\|data) | | BF_incl_ | |
| Condition_framing |  | 0.600 |  | 0.400 |  | 0.154 |  | 0.846 |  | 0.121 |  |
| Condition_format |  | 0.600 |  | 0.400 |  | 0.347 |  | 0.653 |  | 0.354 |  |
| Condition_framing ✻  Condition_format |  | 0.200 |  | 0.800 |  | 0.015 |  | 0.985 |  | 0.062 |  |
|  | | | | | | | | | | | |

***Group differences in pain perception***

Pain threshold (32-51°C)

**Figure S1.** Results for post-treatment group differences in pain threshold, while controlling for baseline pain threshold.

Pain intensity (0-100)

**Figure S2.** Results for post-treatment group differences in in the intensity ratings relating to pain threshold, while controlling for baseline intensity ratings.

**Study 3**

***Bayesian ANOVA for side effect experiences***

Table S3

| *Analysis of Effects – Itching in the Last Few Minutes* | | | | | | | | | | | |
| --- | --- | --- | --- | --- | --- | --- | --- | --- | --- | --- | --- |
| Effects | | P(incl) | | P(excl) | | P(incl\|data) | | P(excl\|data) | | BF_incl_ | |
| Condition |  | 0.500 |  | 0.500 |  | 0.146 |  | 0.854 |  | 0.171 |  |
|  | | | | | | | | | | | |

**References**

Emerson, N. M., Zeidan, F., Lobanov, O. V., Hadsel, M. S., Martucci, K. T., Quevedo, A. S., Starr, C. J., Nahman-Averbuch, H., Weissman-Fogel, I., & Granovsky, Y. (2014). Pain sensitivity is inversely related to regional grey matter density in the brain. *PAIN®*, *155*(3), 566–573.

Kube, T., Rief, W., Vivell, M. B., Schäfer, N. L., Vermillion, T., Körfer, K., & Glombiewski, J. A. (2020). Deceptive and Nondeceptive Placebos to Reduce Pain: An Experimental Study in Healthy Individuals. *The Clinical Journal of Pain*, *36*(2), 68–79. https://doi.org/10.1097/AJP.0000000000000781

Locher, C., Nascimento, A. F., Kirsch, I., Kossowsky, J., Meyer, A., & Gaab, J. (2017). Is the rationale more important than deception? A randomized controlled trial of open-label placebo analgesia. *PAIN*, *158*(12), 2320–2328.
